# Dopant-localized mechanoluminescence in simple oxides

**DOI:** 10.1038/s41377-026-02368-5

**Published:** 2026-06-26

**Authors:** Ankoji Parvathala, Soo Ha Lee, Hong In Jeong, Hyosung Choi

**Affiliations:** Department of Chemistry, Research Institute for Convergence of Basic Science and Research Institute for Natural Sciences, Hanyang University, Seoul, 04763 Republic of Korea

**Keywords:** Optical materials and structures, Micro-optics

## Abstract

Recent work on Al_2_O_3_:Cr^3+^ introduces a compelling mechanoluminescence platform based on a simple oxide, highlighting dopant-localized emission driven by stress-induced carrier dynamics at Cr centers. This advances a shift from bulk-mediated processes to dopant-centered carrier dynamics, enabling more rational and precise materials design. However, the origin of carrier separation required for dopant ionization remains to be clarified, particularly regarding the role of interfacial and heterojunction-induced fields; addressing these aspects may rapidly establish a more comprehensive understanding of the coupled mechanisms involving localized excitation and field-assisted carrier modulation.

Mechanoluminescence (ML) provides a unique route for direct mechano-to-photon conversion, yet its fundamental mechanism remains unresolved^1,2^. Conventional interpretations, including piezoelectric-^3^, triboelectric-^4–6^, and trap-mediated models^7^, have largely described ML as a bulk-mediated carrier process. While these frameworks capture macroscopic emission behavior, they do not fully resolve the microscopic processes governing carrier activation and recombination at local luminescent centers.

The recent investigation by Z. Fang et al. demonstrates intense, self-recoverable ML in a centrosymmetric oxide, Al_2_O_3_:Cr^3+^, where the isolation of Cr-centered dopant sites within a structurally simple lattice provides a direct microscopic pathway for carrier activation and recombination^8^. The study combines density functional theory calculations with experimental validation to reveal that the ML process is fundamentally governed by a stress-driven ionization mechanism at localized dopant sites (Cr^3+^→Cr^4+^-like).

Under stress-free conditions, the active Cr_Al_ defect remains thermodynamically stabilized in a neutral state (Cr_Al⁰_). Upon the application of mechanical strain, however, localized band bending accompanied by enhanced dipole moments at the distorted Cr-centered site induces a valence transition to a positively charged state (Cr_Al⁺_), resulting in the ionization of localized electrons. The release of external stress subsequently enables rapid detrapping of these carriers, followed by radiative recombination at the luminescent center. The released electrons are then efficiently recaptured by the active sites, transiently populating the excited state before relaxing to the ground state, producing intense near-infrared emission (^2^E → ^4^A_2_). This process can be interpreted as a localized piezoelectric-like response, where structural distortion at the Cr-centered octahedral site generates a transient electromechanical field that drives carrier ionization and recombination, as schematically illustrated in Fig. 1.

**Fig. 1:**
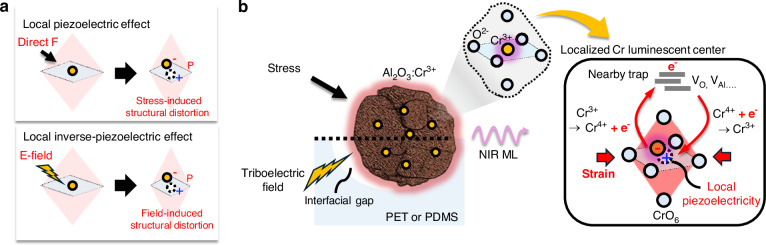
Mechanism of dopant-localized ML in Al_2_O_3_:Cr^3+^. **a** Mechanical stress induces local structural distortion of the Cr-centered octahedral site, generating a localized electromechanical field through symmetry breaking (top). An externally applied electric field induces analogous structural distortion via an inverse electromechanical response at the same defect site (bottom). **b** Dopant-localized ML mechanism in Al_2_O_3_:Cr^3+^. Structural distortion at the Cr-centered site facilitates field-assisted ionization, where a carrier is transiently released and captured by nearby defect-localized trap states, followed by recapture at the luminescent center, leading to radiative recombination

This study is significant from both fundamental and practical perspectives. The authors report unprecedented, triggerable, and highly repeatable ML emissions over ~7000 mechanical loading cycles without any optical pre-irradiation. More importantly, by explicitly linking mechanical strain to dopant-specific valence transitions and transient carrier ionization, this work establishes a concrete microscopic framework for stress-driven carrier modulation. This perspective reinforces the emerging view that ML is governed by carrier dynamics localized at dopant–defect environments rather than by purely bulk-mediated processes^9,10^.

However, this interpretation raises a fundamental question regarding the stress-induced driving force required for Cr dopant ionization. Al_2_O_3_ is a centrosymmetric, wide-bandgap ( ~ 8.8 eV) insulator^11^, which inherently limits bulk piezoelectric responses and band-bending-driven carrier separation^12^. This suggests that Cr ionization at local luminescent centers is likely governed by defect-localized structural distortion or externally induced electric fields^9,13^, with the reported strain-induced “band bending” more appropriately interpreted as a localized electronic perturbation at Cr-centered defect sites rather than a macroscopic band modulation within the bulk Al_2_O_3_ lattice. Furthermore, given the PET(or PDMS)-Al_2_O_3_ configuration in this work, interfacial triboelectric effects may contribute to carrier activation given the known triboelectric interactions at polymer–oxide interfaces^14,15^, necessitating a clear distinction from triboelectric-induced structural distortion or band bending, highlighting the need to distinguish intrinsic defect-driven processes from interfacial field-assisted effects^13^.

Based on the dopant-localized mechanism described above, the role of the Al_2_O_3_/Ga_2_O_3_ heterojunction, which introduces band offsets within the wide bandgap of Al_2_O_3_, remains mechanistically unclear^16–18^. If ML is governed predominantly by ionization at localized Cr centers, the relevance of band alignment in a wide-bandgap insulating Al_2_O_3_ becomes less direct unless mesoscale carrier redistribution, potentially involving trap-mediated host–dopant charge transfer, contributes to the process. This suggests that the role of the heterojunction may extend beyond simple band-offset effects, pointing toward a more complex energy transfer interplay.

In this scenario, interfacial bonding such as Al–O–Ga linkages can form at the Al_2_O_3_/Ga_2_O_3_ heterojunction due to the chemical compatibility of the oxide framework. Such interfacial configurations can locally break symmetry and generate electromechanical responses under mechanical stress or interfacial triboelectric fields (e.g., in PET-Al_2_O_3_ systems)^19–21^. These locally enhanced fields may facilitate field-induced ionization at Cr centers or promote strain-coupled structural distortion, ultimately contributing to the observed enhancement in ML intensity^22^.

Taken together, this work represents a significant step toward redefining ML in terms of dopant-localized carrier dynamics in simple oxide systems. At the same time, it suggests that ML may be governed by a coupled interplay between defect-localized processes and extrinsic field-assisted mechanisms. Establishing such a unified microscopic framework will be essential for the rational design of next-generation mechanoluminescent materials.
